# Retracted: Application Effect of Different Concentrations of Platelet-Rich Plasma Combined with Quadriceps Training on Cartilage Repair of Knee Osteoarthritis

**DOI:** 10.1155/2022/9839871

**Published:** 2022-11-25

**Authors:** Journal of Healthcare Engineering

*Journal of Healthcare Engineering* has retracted the article titled “Application Effect of Different Concentrations of Platelet-Rich Plasma Combined with Quadriceps Training on Cartilage Repair of Knee Osteoarthritis” [1] due to concerns that the peer review process has been compromised.

Following an investigation conducted by the Hindawi Research Integrity team [2], significant concerns were identified with the peer reviewers assigned to this article; the investigation has concluded that the peer review process was compromised. We therefore can no longer trust the peer review process, and the article is being retracted with the agreement of the Chief Editor.

## References

[1] Wu Z., Yin J., Yue Y., Zhang Y. (2022). Application Effect of Different Concentrations of Platelet-Rich Plasma Combined with Quadriceps Training on Cartilage Repair of Knee Osteoarthritis. *Journal of Healthcare Engineering*.

[2] Ferguson L. (2020). Advancing Research Integrity Collaboratively and with Vigour. https://www.hindawi.com/post/advancing-research-integrity-collaboratively-and-vigour/.

